# Generative Artificial Intelligence in Urology: Navigating the Frontier of Ethical, Legal, and Clinical Challenges

**DOI:** 10.7759/cureus.97047

**Published:** 2025-11-17

**Authors:** Waqas Khalil, Mazhar Sheikh, Jawad U Islam

**Affiliations:** 1 Urology, Blackpool Teaching Hospitals, Blackpool, GBR

**Keywords:** artificial intelligence, artificial intelligence in medicine, artificial intelligence in surgery, endourology, urology, urology department

## Abstract

The emergence of generative artificial intelligence (AI), particularly large language models and image-generation tools, is poised to transform the field of urology. These technologies enable innovative applications in medical education, clinical decision support, patient communication, and surgical planning, extending beyond traditional analytical AI by creating new content, from synthetic clinical notes to simulated surgical environments. In urology, these capabilities translate into automated summarization of complex patient histories, generation of patient-specific three-dimensional anatomical reconstructions, and support for differential diagnosis in conditions such as prostate cancer or renal masses. However, the rapid adoption of generative AI also introduces significant ethical, legal, and clinical challenges. Risks are amplified in urological practice, where sensitive imaging data, biomarker profiles, and diagnostic decision pathways may be vulnerable to privacy breaches, algorithmic bias, or erroneous AI-generated recommendations. Hallucinated outputs, such as incorrect treatment summaries or misinterpreted radiologic features, can directly compromise patient safety if not rigorously validated. This review synthesizes the current landscape of generative AI in urology, critically examines these discipline-specific risks, and proposes a structured framework for responsible integration into clinical workflows. We highlight the need for transparent governance, bias mitigation, prospective validation, and interdisciplinary collaboration to ensure that generative AI enhances, rather than undermines, the quality, equity, and safety of urologic care.

## Introduction and background

Artificial intelligence (AI) has progressively become integrated into the fabric of modern medicine, providing powerful tools for diagnostic imaging, predictive analytics, and personalized treatment planning [1]. In urology, traditional (“discriminative”) AI models have demonstrated high proficiency in tasks such as detecting prostate cancer on multiparametric MRI, segmenting tumors, predicting renal stone composition, and optimizing robotic surgical maneuvers [2,3]. The recent advent of generative AI, a subset of AI capable of creating novel text, images, and other data, marks a paradigm shift. Technologies such as large language models (LLMs) (e.g., GPT-4, Bard) and image-generating models (e.g., DALL-E, Midjourney) introduce capabilities that extend beyond analysis to content creation [4].

Unlike earlier AI systems, generative AI has already begun to show concrete value within urology. For example, recent studies have demonstrated the successful use of LLMs in generating first drafts of operative notes for endourological procedures, improving documentation speed and consistency (pilot evaluations reported in academic urology settings). Similarly, proof-of-concept work in prostate cancer imaging has used generative adversarial network-based synthetic MRI to augment training datasets, improving lesion detection performance in biopsy-proven cases. These early applications highlight not only feasibility but also the discipline-specific utility of generative models.

In urology, potential applications are vast and include the automated generation of clinical notes, summarization of complex patient records, creation of personalized educational materials for patients, and the development of highly realistic surgical simulations for training [5]. Published examples include LLM-assisted patient counselling scripts for benign prostatic hyperplasia and AI-generated three-dimensional (3D) reconstructions used in planning partial nephrectomy, demonstrating early but tangible clinical relevance. Despite this promising potential, the deployment of generative AI is not without peril. The “black box” nature of many models, their dependence on potentially biased training data, and the risk of generating confident misinformation pose significant ethical, legal, and clinical dilemmas [6,7].

Importantly, a clear gap exists in the current literature: while generative AI has rapidly expanded across radiology, oncology, and pathology, its implications for urology remain fragmented and under-characterized. Urology is uniquely positioned for generative AI transformation because it sits at the intersection of imaging-heavy diagnostics, minimally invasive and robot-assisted surgery, and chronic disease management domains where generative models can directly enhance interpretation, simulation, and patient engagement. Yet, no consolidated framework currently exists to evaluate the ethical, legal, and clinical challenges specific to urological care.

To address this gap, this review adopts a narrative review methodology, synthesizing current evidence and expert perspectives across clinical, educational, and surgical domains. This approach is appropriate given the novelty of the field and the heterogeneity of available studies. The aim is to explore the burgeoning applications of generative AI within urology, critically analyze the associated challenges, and propose a roadmap for its conscientious and responsible adoption.

## Review

Expansive applications of generative AI in urology

Medical Education and Training

The integration of generative AI into urological education represents a major evolution from passive, text-based learning to interactive, adaptive, and simulation-rich environments. By generating dynamic content that mirrors real-world clinical variability, these technologies offer individualized and high-fidelity training opportunities that were previously unattainable.

Synthetic Surgical Scenarios

Generative AI can construct diverse and anatomically accurate virtual surgical cases tailored to urology. For example, AI-driven simulators can recreate variations of robotic-assisted radical prostatectomy complicated by unexpected venous bleeding or simulate a partial nephrectomy involving a complex hilar tumor. These dynamic models allow residents to rehearse intraoperative decision-making, refine psychomotor skills, and build confidence in managing rare or high-stakes events, all within a safe, zero-risk environment [8,9]. Such exposure has the potential to accelerate skill acquisition and decrease the learning curve associated with technically demanding procedures.

Personalized Learning Pathways

Beyond static educational materials, generative AI can analyze a trainee’s performance across simulations, quizzes, or structured assessments to identify specific gaps in knowledge or technique. The system can then automatically curate individualized learning modules, directing residents to targeted content in subspecialties such as neuro-urology, uro-oncology, or pediatric urology [10]. This data-driven personalization transforms training from a one-size-fits-all curriculum to a competency-based, adaptive model aligned with modern medical education standards.

Interactive Socratic Tutoring

LLMs can serve as highly responsive educational tutors. Through natural language interaction, they can answer advanced questions on pathophysiology, surgical technique, and postoperative management, mimicking a Socratic teaching style. These systems can generate realistic oral-board practice questions, provide immediate mechanistic explanations, and adjust the complexity of responses based on learner proficiency. This creates an accessible and continuously available teaching resource that supplements faculty instruction and enhances self-directed learning.

Clinical decision support and documentation

Generative AI holds considerable potential in clinical decision-making and administrative workflows, areas that directly affect patient care quality, clinician efficiency, and resource utilization.

Automated Literature Synthesis

Managing complex cases often requires a rapid synthesis of evolving evidence. Generative AI systems can summarize clinical trials, guidelines, and systematic reviews relevant to scenarios such as metastatic castration-resistant prostate cancer, offering a concise, evidence-based overview within seconds [11]. This capability significantly reduces the time clinicians spend navigating vast medical literature and provides near-instant updates in fast-moving therapeutic areas.

Drafting Clinical Documentation

AI-assisted drafting of clinical notes, including consult summaries, operative reports, and discharge instructions, can substantially reduce administrative burden. Using structured electronic health record (EHR) inputs or voice transcripts, generative models produce draft documentation that clinicians can then refine [12]. When paired with robust oversight and final physician review, these tools can improve documentation consistency and free up valuable time for direct patient care, potentially mitigating burnout.

Patient communication and engagement

Generative AI has the capacity to enhance patient-centered care by bridging gaps in health literacy, improving communication across languages, and personalizing educational materials.

Demystifying Medical Jargon

LLMs can translate technical urological terminology, such as hematuria, benign prostatic hyperplasia, or cystoscopy, into accessible, patient-friendly language. This enables patients to better understand their conditions, improves treatment adherence, and reduces anxiety by fostering clarity and transparency during counseling.

Multilingual and Accessibility Support

AI-powered translation tools can facilitate clear communication with patients who have limited proficiency in the clinician’s primary language. By providing accurate real-time translation of educational handouts, consent forms, and postoperative instructions, these technologies promote equitable care for linguistically diverse populations [13].

Generating Personalized Information Sheets

Generative AI can tailor educational materials to reflect a patient’s specific diagnosis, comorbidities, treatment plan (e.g., BCG therapy for bladder cancer), and even preferred reading level. Such personalized content supports shared decision-making and strengthens patient engagement across the continuum of care.

Surgical planning, robotics, and intraoperative assistance

Urology, as one of the most technology-integrated surgical specialties, stands to benefit greatly from generative AI innovations that enhance preoperative planning, operative efficiency, and real-time decision support.

Patient-Specific Preoperative Simulation

Using CT or MRI data, generative models can produce high-resolution, patient-specific 3D anatomical reconstructions. These reconstructions help surgeons visualize tumor boundaries, vascular anatomy, and potential anatomical variations during procedures such as partial nephrectomy for complex renal masses [14]. Enhanced preoperative planning can lead to more precise dissection, reduced intraoperative complications, and improved functional outcomes.

Workflow Optimization and Predictive Analytics

By analyzing historical surgical videos, EHR data, and robotic console metrics, AI can predict case duration, anticipate instrument needs, and identify potential intraoperative bottlenecks. These predictive insights allow for optimized operating room scheduling, improved resource allocation, and reduced operative delays, factors that contribute to both clinical efficiency and cost-effectiveness.

Intraoperative Guidance

Emerging research is exploring real-time generative overlays for robotic-assisted procedures. These systems can project crucial information, such as tumor margins, vessel locations, or nerve pathways, directly onto the surgeon’s visual field during robotic surgery, functioning as an augmented reality guide [15]. This integration could support greater precision, reduce the risk of inadvertent injury, and enhance the surgeon’s situational awareness.

Critical ethical challenges

Accountability and Liability

A primary ethical concern is determining responsibility when an AI-generated recommendation contributes to an adverse clinical outcome. Questions arise regarding whether accountability lies with the urologist who applied the recommendation, the institution that implemented the tool, or the developers who designed the algorithm. The principle of a “human-in-the-loop” remains essential, ensuring that the treating urologist retains ultimate legal and ethical responsibility for verifying AI-generated information and making final clinical decisions [7]. Developing clear medicolegal frameworks is therefore necessary to define responsibilities across clinicians, institutions, and AI developers in this evolving landscape.

Bias and Fairness

Generative AI models are trained on large datasets that may insufficiently represent certain demographic or clinical subgroups. When training data are disproportionately derived from Caucasian populations, model outputs, such as interpretations of PSA trends or treatment recommendations for prostate cancer, may be less accurate for patients of other racial or ethnic backgrounds [16]. Such biases risk reinforcing existing disparities in urologic care. Continuous auditing for bias in both the underlying data and model outputs is essential before deployment and throughout clinical use to ensure equitable performance.

Transparency and Explainability

Many advanced generative models operate as “black boxes,” producing outputs without providing insight into the reasoning behind them. For urologists to confidently incorporate AI-derived guidance, such as treatment recommendations for muscle-invasive bladder cancer, they must understand the factors influencing the model’s conclusions. The field of Explainable AI (XAI) is therefore critical, as it aims to produce models capable of clarifying which patient features, clinical variables, or evidence sources informed a given recommendation [6]. Improved explainability is essential for clinical trust and safe integration.

Patient Consent and Privacy

Generative AI often requires access to sensitive patient information, raising important concerns about transparency and data protection. Patients have the right to understand when AI systems are being used, how their data contributes to AI-assisted documentation or decision support, and what safeguards are in place [17]. Informed consent processes must evolve to include explicit disclosure of AI involvement in clinical workflows. At the same time, strong data anonymization, encryption, and security protocols are necessary to prevent breaches, particularly when cloud-based AI solutions are employed.

Legal and regulatory considerations

Regulatory Oversight

Regulatory agencies such as the U.S. Food and Drug Administration (FDA) and the European Medicines Agency are extending existing software-as-a-medical-device frameworks to accommodate generative AI. A central challenge lies in overseeing adaptive algorithms that continue to learn after deployment, rendering traditional one-time pre-market approvals insufficient. This shift necessitates continuous performance evaluation, post-market surveillance, and robust mechanisms for detecting model drift. The FDA’s proposed “Predetermined Change Control Plan” exemplifies an emerging strategy for regulating ongoing algorithm modifications and ensuring that safety and efficacy are maintained over time [18].

Intellectual Property and Authorship

The rise of generative AI introduces complex questions regarding ownership and authorship. Current copyright laws generally require human creativity, complicating claims over AI-generated operative notes, synthetic imaging, or AI-assisted academic content. Similar challenges emerge when AI tools contribute substantially to manuscripts, guidelines, or educational materials. Professional organizations and academic publishers increasingly mandate disclosure of AI involvement, emphasizing that while AI may support content development, it cannot be recognized as a legal author and that human researchers must retain full responsibility for the final work [19].

Institutional Policies and Data Governance

Healthcare institutions play a critical role in establishing safe and responsible AI use. Effective governance requires clearly defined clinical use cases, standardized EHR integration pathways, and stringent data security measures that address access control, auditability, and information stewardship. Many centers are adopting multidisciplinary oversight committees that bring together expertise from clinical urology, information technology, cybersecurity, legal affairs, and bioethics to guide implementation, evaluate risks, and ensure compliance with regulatory and ethical standards. Such institutional frameworks are essential for harmonizing innovation with patient safety and legal accountability.

Clinical safety and practical implementation concerns

Misinformation and Hallucinations

Generative AI systems may produce confident but inaccurate outputs, a phenomenon known as hallucination. In urology, such errors can have significant clinical consequences; for example, if an AI-generated summary misstates chemotherapy dosing recommendations or fabricates evidence from nonexistent studies. Because of this inherent risk, all AI-derived clinical content must be independently verified against authoritative, up-to-date sources by the treating clinician before it informs decision-making [11]. Ensuring a robust verification step is essential to prevent patient harm and maintain clinical reliability.

Deskilling and Overreliance

Increased automation raises the concern that clinicians may gradually lose essential skills if they rely too heavily on AI for routine cognitive tasks. Dependence on automated documentation, literature synthesis, or preliminary diagnostic reasoning could erode a urologist’s clinical judgment, medical knowledge, and communication abilities over time. The clinician’s role must therefore remain that of an expert evaluator, interpreting, validating, and contextualizing AI outputs rather than passively accepting them. Maintaining active engagement with core clinical tasks is crucial for preserving human expertise.

Workflow Integration and Clinical Adoption

AI tools that are poorly integrated into clinical workflows can impede rather than streamline care. Challenges such as unintuitive interfaces, excessive alerts, and incompatibility with existing EHR systems may contribute to inefficiency, clinician frustration, and eventual rejection of the technology. Successful implementation requires thoughtful, user-centered design and continuous involvement of practicing urologists in the development, testing, and refinement phases. When aligned with real-world clinical processes, AI systems are more likely to be accepted, trusted, and effectively utilized in patient care.

Future directions

The future integration of generative AI in urology will depend on the establishment of clear, specialty-specific standards and the preparation of clinicians to work effectively with these technologies. Professional societies such as the American Urological Association and European Association of Urology should develop guidelines that define appropriate use cases, expected levels of accuracy, and safeguards addressing fairness, validation, and transparency. Equally important is the incorporation of AI literacy into residency curricula and continuing medical education, ensuring that urologists understand the capabilities and limitations of generative models and can critically evaluate their outputs in clinical settings. Rigorous prospective validation of AI tools in real-world urological workflows, followed by continuous performance monitoring, is essential to ensure safety, reliability, and clinical relevance.

Successful implementation will also require coordinated oversight and a commitment to patient-centered design. Interdisciplinary “AI-stewardship” teams, including urologists, data scientists, ethicists, and legal experts, should guide the evaluation, deployment, and auditing of these systems within institutions. Above all, the aim must be to ensure that generative AI augments rather than replaces clinical judgment, strengthens communication, and reduces administrative burden. When developed with these priorities in mind, generative AI has the potential to enhance decision-making, improve surgical planning, and, ultimately, support more efficient and compassionate urologic care.

## Conclusions

Generative AI stands at the frontier of a new era in urology, offering unprecedented tools to advance education, clinical care, and surgical innovation. However, its transformative potential is inextricably linked with profound ethical, legal, and clinical challenges. Navigating this complex landscape requires a proactive, cautious, and principled approach. By establishing robust governance, prioritizing transparency and fairness, and maintaining the central role of the urologist as the ultimate clinical decision-maker, the field can harness the power of generative AI to improve patient outcomes while upholding the highest standards of safety, equity, and professional responsibility. The future of urology will undoubtedly be shaped by AI, but it must be a future guided by human wisdom, oversight, and an unwavering commitment to ethical practice.

## References

[1] Topol EJ (2019). High-performance medicine: the convergence of human and artificial intelligence. Nat Med.

[2] Liu X, Faes L, Kale AU (2019). A comparison of deep learning performance against health-care professionals in detecting diseases from medical imaging: a systematic review and meta-analysis. Lancet Digit Health.

[3] Jha S, Topol EJ (2016). Adapting to artificial intelligence: radiologists and pathologists as information specialists. JAMA.

[4] Rajpurkar P, Chen E, Banerjee O, Topol EJ (2022). AI in health and medicine. Nat Med.

[5] Meskó B, Drobni Z, Bényei É, Gergely B, Győrffy Z (2017). Digital health is a cultural transformation of traditional healthcare. Mhealth.

[6] Hamet P, Tremblay J (2017). Artificial intelligence in medicine. Metabolism.

[7] Gerke S, Minssen T, Cohen G (2020). Ethical and legal challenges of artificial intelligence in healthcare. Artificial Intelligence in Healthcare.

[8] Yi X, Walia E, Babyn P (2019). Generative adversarial network in medical imaging: a review. Med Image Anal.

[9] Hashimoto DA, Rosman G, Rus D, Meireles OR (2018). Artificial intelligence in surgery: promises and perils. Ann Surg.

[10] Park SH, Do KH, Kim S, Park JH, Lim YS (2019). What should medical students know about artificial intelligence in medicine?. J Educ Eval Health Prof.

[11] Vaishya R, Misra A, Vaish A (2023). ChatGPT: is this version good for healthcare and research?. Diabetes Metab Syndr.

[12] Senders JT, Arnaout O, Karhade AV, Dasenbrock HH, Gormley WB, Broekman ML, Smith TR (2018). Natural and artificial intelligence in neurosurgery: a systematic review. Neurosurgery.

[13] Jawetz ST, Fox MG, Blankenbaker DG (2023). ACR Appropriateness Criteria® Chronic Hip Pain: 2022 update. J Am Coll Radiol.

[14] Hung AJ, Chen J, Che Z (2018). Utilizing machine learning and automated performance metrics to evaluate robot-assisted radical prostatectomy performance and predict outcomes. J Endourol.

[15] Mascagni P, Vardazaryan A, Alapatt D (2022). Artificial intelligence for surgical safety: automatic assessment of the critical view of safety in laparoscopic cholecystectomy using deep learning. Ann Surg.

[16] Obermeyer Z, Powers B, Vogeli C, Mullainathan S (2019). Dissecting racial bias in an algorithm used to manage the health of populations. Science.

[17] Price WN 2nd, Cohen IG (2019). Privacy in the age of medical big data. Nat Med.

[18] (2025). U.S. Food and Drug Administration. Artificial intelligence and machine learning (AI/ML)-enabled medical devices. https://www.fda.gov/medical-devices/software-medical-device-samd/artificial-intelligence-enabled-medical-devices.

[19] Hosseini M, Rasmussen LM, Resnik DB (2024). Using AI to write scholarly publications. Account Res.

